# Ethyl Pyruvate: An Anti-Microbial Agent that Selectively Targets Pathobionts and Biofilms

**DOI:** 10.1371/journal.pone.0162919

**Published:** 2016-09-22

**Authors:** Tewodros Debebe, Monika Krüger, Klaus Huse, Johannes Kacza, Katja Mühlberg, Brigitte König, Gerd Birkenmeier

**Affiliations:** 1 Institute of Medical Microbiology, University of Leipzig, Leipzig, Germany; 2 Institute of Biochemistry, Faculty of Medicine, University of Leipzig, Leipzig, Germany; 3 Institute of Bacteriology and Mycology, University of Leipzig, Leipzig, Germany; 4 Leibniz Institute on Aging—Fritz Lipmann Institute, Jena, Germany; 5 Institute of Veterinary Anatomy, Faculty of Veterinary Medicine, University of Leipzig, Leipzig, Germany; 6 Department of Internal Medicine, Neurology & Dermatology, Angiology, University of Leipzig, Leipzig, Germany; 7 College of Medicine and Health Sciences, Bahir Dar University, Bahir Dar, Ethiopia; Universite Paris-Sud, FRANCE

## Abstract

The microbiota has a strong influence on health and disease in humans. A causative shift favoring pathobionts is strongly linked to diseases. Therefore, anti-microbial agents selectively targeting potential pathogens as well as their biofilms are urgently demanded. Here we demonstrate the impact of ethyl pyruvate, so far known as ROS scavenger and anti-inflammatory agent, on planktonic microbes and biofilms. Ethyl pyruvate combats preferably the growth of pathobionts belonging to bacteria and fungi independent of the genera and prevailing drug resistance. Surprisingly, this anti-microbial agent preserves symbionts like Lactobacillus species. Moreover, ethyl pyruvate prevents the formation of biofilms and promotes matured biofilms dissolution. This potentially new anti-microbial and anti-biofilm agent could have a tremendous positive impact on human, veterinary medicine and technical industry as well.

## Introduction

Undoubtedly, antibiotics have significantly improved human health and life expectancy. Nonetheless, we have to keep in mind that antibiotics may lead to a perturbation of the existing physiological/beneficial microbiota balance which often results in the emergence of potentially pathogenic microbes, so called pathobionts. It is now well accepted that a disturbed gut microbiota is a main reason for an increased susceptibility to subsequent chronic diseases such as adiposity, metabolic syndrome, inflammatory diseases, cancer and neurological disorders [1–3]. Moreover, a disturbed vaginal microbiota during pregnancy seems, e.g. through the use of antibiotics or hormonal changes, to be responsible or at least to attribute for preterm birth and to influence the development of the neonate immune system and the susceptibility for chronic diseases including obesity [4].

The ability of microbes to form a biofilm on biological as well as on non-biological surfaces, a highly structured community of microbes encased in a self-produced protective extracellular matrix, presents another great challenge in medicine and industry [5]. In this regard, biofilm-associated infections are notoriously resistance to both conventional antimicrobial agents and host immune system [6]. Biofilm-associated microorganisms show a 100 to 1,000-fold increase in anti-microbial tolerance compared with planktonic cells [7] and have important negative effects on human health. Examples are *Pseudomonas aeruginosa* infections in cystic fibrosis, *Staphylococcus epidermidis*- and *Staphylococcus aureus*-related implant infections, recurrent urinary tract infections, and periodontal diseases [8]. In summary, antibiotic tolerance mechanisms in biofilms include failure of antibiotics to penetrate biofilms, a slow growth rate, an altered metabolism, the appearance of persister cells, an extracellular biofilm matrix and Quorum-sensing.

The emergence and spread of microorganisms resistant to multiple anti-microbial agents is another great challenge to the medicine and a serious threat to global public health affecting all parts of the world. However, while resistance rates continue to rise, the rate of antibiotic discovery has dropped substantially. Sadly, only a few new agents have recently been approved and are available [9]. Therefore, there is an urgent need for new approaches in the field of anti-microbial therapy, having the capacity to eradicate biofilms as well.

Ethyl pyruvate (EP), a simple ester of pyruvate, seems to meet such a demand. First notations of biological effects of EP came from studies showing its capability to ameliorate intestinal or hepatic injury during experimental ischemia, reperfusion and endotoxemia by acting as a ROS scavenger and suppressor of pro-inflammatory cytokines. Many putative targets of EP have been supposed to explain its beneficial effects in mammalian cells [10]. Recently, we have shown that EP effectively inhibits the growth of cells relying predominantly on glycolysis [11]. Its auspicious anti-tumor [12] and anti-trypanosome activity [13] corroborate the assumption that EP directly interferes with glucose metabolism. Target analysis revealed that EP inhibits enzymes of the glycolytic and para-glycolytic pathway, such as the glyoxalase 1 (GLO 1), and pyruvate kinase (PK) [13, 14]. Accordingly, EP causes depletion of cellular ATP and affects mitochondrial oxidation via induced accumulation of the toxic methylglyoxal, respectively [11].

Against this background we deduced that many of those cells and microbes relying mainly on glucose oxidation should be sensible to EP treatment. Therefore, we launched a comprehensive study to investigate the effect of EP on (i) a large number of different aerobic and anaerobic pathobionts with clinical significance including yeast cells and fungi; (ii) on pathobionts with specific resistance mechanisms and (iii) on mature and developing biofilms.

**Fig 1:**
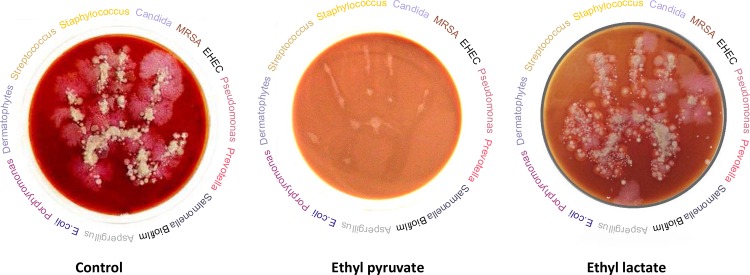
Graphical abstract of anti-microbial activity of ethyl pyruvate. Fingerprints of non-disinfected hands on blood agar containing ethyl pyruvate (middle) ethyl lactate (right) and medium (left). The microbes displayed at the circumferences of the agar plates depict a selection of microbes investigated in the present study. Plates were incubated at 37°C for 48 h.

Here, we display new properties of EP as pathobiont-selective anti-microbial activity with an additional high potential as a novel anti-biofilm agent. In this regard we present evidence that EP has anti-microbial properties against both planktonic cells and biofilms of pathobionts and against microorganisms with clinical relevant resistance. Advantageously, EP shows reduced anti-microbial activities against symbiotic microorganisms such as *Lactobacillus species*. Surprisingly, ethyl lactate (EL) that differs from EP by two protons only is lacking anti-microbial activities at all (Fig 1).

## Materials and Methods

### Microorganisms and growth condition

Clinical isolates of *Candida spp*. were maintained on Sabouraud dextrose agar (Oxoid, Wesel, Germany) and incubated at 37 ⁰C for 24 h. *Lactobacillus spp*. were maintained on de Man Rogosa Sharpe (MRS) agar (Oxoid) and blood agar (Carl Roth GmbH, Karlsruhe, Germany), followed by incubation at 37 ⁰C, 5% CO_2_ for 24–48 h. Gram-negative bacteria, particularly *Escherichia coli* were maintained on blood agar and incubated at 37 ⁰C for 24 h. Subsequently, species level identification was done using the Matrix-assisted laser desorption ionization time-of-flight (MALDI-TOF) mass spectrometry (bioMérieux, Marcy I’Etoile, France). All identified microbes were stored at -80°C in a preservative Cryobank tubes (CRYOBANK^TM^ Mast Group Ltd., Merseyside, UK) according to the manufacturer’s instruction. All strains were isolated from clinical specimens.

### MALDI-TOF-MS based microbe identification

The automated MALDI-TOF was performed following standard protocol (bioMérieux, Marcy I’Etoile, France). Freshly growing pure microbial cells and control cells (*Escherichia coli*) were directly analyzed (VITEK®-MS, bioMérieux, Marcy I’Etoile, France). The sample spectra were compared to an extensive database of spectra from bacterial species by VITEK®-MS proofing, which allow us to accurately identify the microorganism in question.

### Determination of the Minimum Inhibitory Concentration of Candida species

Minimum Inhibitory Concentrations (MIC) of EP were determined for planktonic *Candida spp*. using sterile flat-bottom 96-well microtiter plates (Cellstar® Greiner Bio-One, Frickenhausen, Germay) according to EUCAST guideline [15]. The RPMI-1640 medium (Sigma-Aldrich, Life science) supplemented with glutamine, and glucose to a final concentration of 20 g/L, pH 7.0, was sterilized using a 0.22-μm filter (TPP^®^, Techno Plastic Products, Trasadingen, Switzerland). Serial two-fold dilutions of the EP were prepared in this medium in the 96-well plate to obtain a final concentration range of 0.39 to 200 mM. Subsequently, 100 μL standardized working yeast suspension was inoculated into each column containing the EP and growth control column, which provided the required final inoculum density of 0.5–2.5 x 10^5^ CFU/mL. Afterward, the plates were incubated at 37°C for 24 h. Growth inhibition was determined visually and confirmed by spectrophotometry at 600 nm by a microdilution plate reader (Anthos htIII Microsystem, Krefeld, Germany). The lowest concentration at which there was no turbidity or growth compared to growth in the EP-free well (growth control) was regarded as Minimum Inhibitory Concentration (MIC) of the compound. Quality control was done by taking column 11 as a growth control and column 12 as a sterility control (n = 3).

### Determination of the Minimum Inhibitory Concentration of bacteria

The MICs of EP against bacteria isolates were determined on a sterile, round-bottom 96-well microtiter plate (Greiner Bio-One, Frickenhausen, Germany) as described by [16, 17] with minor modifications. Serial two-fold dilutions of the EP were prepared in standardized LAB susceptibility testing medium (LSM) broth formulation, essentially consisting of a mixture of Iso-Sensitest (ISO) broth (90%) and (MRS) broth (10%) (Oxoid) adjusted to pH 6.7 for *Lactobacillus spp*. and Müller Hinton broth for *Escherichia coli* to obtain the final concentration ranging from 0.39 to 200 mM. Subsequently, 50 μL standardized working bacterial suspension was inoculated into each column containing the EP and growth control, which provided the required final inoculum density of 5 x 10^5^CFU/mL. A volume of 100 μL of medium was transferred into column 12 as sterility control. Afterward, the plates were incubated at 37°C for 24 h for *Escherichia coli* or at 37°C, 5% CO_2,_ for 24–48 h for *Lactobacillus spp*. Growth inhibition was determined visually and confirmed by spectrophotometry at 600 nm by a microdilution plate reader (n = 3).

### Determination of Minimum Bactericidal Concentration and Minimum Fungicidal Concentration

Minimum Bactericidal Concentration (MBC) and Minimum Fungicidal Concentration (MFC) were determined by sub-culturing samples (10 μL) having a value of higher or equal to the MIC value onto Sabouraud dextrose agar for *Candida spp*., blood agar, and MRS agar for *Escherichia coli* and *Lactobacillus spp*., respectively. The highest dilution that yielded no single microbial colony was considered as MBC for bacteria isolates and MFC for yeast isolates (n = 3).

### Agar macrodilution test

To evaluate anti-microbial activity of EP in agar against a wide panel of isolates namely Dermatophytes (obtained from INSTAND e.V., Society for Promoting Quality Assurance in Medical Laboratories, Duesseldorf, Germany), *Candida spp*., and *Aspergillus spp*. and other moulds, *Salmonella spp*., *Campylobacter spp*., Gram-positive cocci, *Escherichia coli*, *Lactobacillus spp*., *Helicobacter pylori*, *Clostridium spp*., Gram-negative oxidative positive and non-spore forming bacteria (all obtained from clinical specimens), media containing EP with different concentrations was prepared. Briefly, for fungal isolates, Sabouraud dextrose agar plates (Sifin, 2% glucose chloramphenicol agar) with different concentrations of EP (0, 1, 5, 10, 20 and 40 mM) were prepared. Afterward, all dermatophytes were first grown on Sabouraud dextrose agar. To ensure a homogeneous microbial suspension for inoculation, the isolates were rubbed with 5% Tween 80 in Sabouraud broth. Thereafter, aliquots of 5 μL of this suspension were inoculated on Sabouraud dextrose agar plates containing the corresponding concentrations of the EP. Subsequently, the plates were incubated at 30°C for 21 days. Standardized bacterial cells suspension were cultured onto Nutrient broth (NB), Tetrathionate broth (TB), blood agar, Brucella broth medium, de Man Rogasa and Sharpe (MRS), Brain Heart Infusion (BHI) with or without EP under aerobic, microaerophilic or anaerobic conditions depending on the microbiological characteristics of tested isolates for 24–120 h. Finally, plates were examined for the growth of tested pathobionts.

### Biofilm formation

To evaluate the effect of ethyl pyruvate (EP) and ethyl lactate (EL) on *Candida albicans* biofilms during the developmental phase, biofilm formation was done in a 96-wells plate. Briefly, overnight grown colonies of *Candida albicans* were transferred into suspension medium (bioMérieux, Marcy I’Etoile, France) and 800 μL of the suspension was transferred into a cuvette and adjusted to an O.D. value of 2 (~ 10^8^ CFU/mL) at 600 nm using a spectrophotometer (Pharmacia Biotech Ultrospec 2000, Cambridge, UK). Subsequently, the yeast cell suspension was adjusted to 1 x 10^6^ CFU/mL in RPMI-1640 medium and seeded onto 96-well plates. Afterward, plates were incubated at 37 ᴼC for 90 min to induce adhesion [18]. After this adhesion phase, medium was aspirated, non-adherent cells were removed and plates were washed by sterile 10 mM PBS (Gibco, Life Technologies, Germany). Following washing, 100 μL of different sub-inhibitory concentrations of EP (0, 0.2, 0.4, 0.5, 0.56, 0.6 and 0.8 x MIC) were prepared in RPMI-1640 medium and transferred into each well containing the pre-washed biofilms. Thereafter, the plates were further incubated at 37 ᴼC for 24–48 h until formation of mature biofilms occurred. To evaluate the effect of EP on pre-formed biofilms, yeast cells were suspended in RPMI-1640 medium, transferred into 96-wells plate and incubated at 37 ᴼC for 24h. Afterward, EP at different concentrations (0.5, 1, 2, 4, and 8xMIC) was added into plates containing matured biofilms and incubated for further 24 h. At each step of the experiment, the adhered biofilms were confirmed by observation using an inverted microscope (Motic AE31, Ted Pella, Inc. CA, USA). Finally, biofilm formation inhibition and destruction were quantified by XTT assay as described below. Similar procedure was implemented for ethyl lactate (EL) and Amphotericin B (AmpB) (n = 3).

### XTT reduction assay

Biofilm formation inhibition and destruction were quantified by the colorimetric XTT reduction assay (Roche Applied Science, Mannheim, Germany) according to the manufacturer’s instruction. Briefly, a mixture of 5 mL of XTT labeling reagent (1mg/mL) and 0.1 mL electron-coupling reagent (1.25 mM; PMS, N-methyldibenzopyrazine methyl sulfate) was prepared. Next, 100 μL of RPMI-1640 medium was transferred into 96-well culture plates followed by addition of 50 μL XTT labeling mixture solution. This was followed by incubation of the plates at 37 ᴼC for 4 h. Finally, the color change of the solution was measured spectrophotometrically at 492 nm with a multimode microplate reader (TECAN, Grödig, Austria).

### Microscopic assay

To evaluate the effect of EP on biofilm formation, *Candida albicans* biofilms were formed on Thermanox^TM^ plastic cover slips (NUNC, Rochester, NY, USA) in a 12-wells plate (Cellstar® Greiner Bio-One, Frickenhausen, Germany) by dispersing 3 mL of 1x 10^6^ cells/mL suspension in RPMI-1640 medium and incubating at 37°C for 90 min to allow cell adherence. Afterward, the plastic cover slips containing the biofilms were transferred to sterile 12-well plate containing 3 mL of RPMI-1640 medium with or without EP. The plates were then incubated at 37°C for further 24 h. Thereafter, cover slips with biofilms were transferred to glass microscopic slides, stained with Calcofluor white solution (Polysciences, Inc, Warrington, USA) and incubated at 25°C for 1 min. Finally, the stained biofilms were examined by a fluorescence microscope with ultraviolet light (excitation 340 to 380 nm with 430 nm suppression) at a magnification of x10 (Leica, Germany). In order to evaluate the effect of EP on the destruction of matured *Candida albicans* biofilms, yeast cells suspended in RPMI-1640 medium were transferred into 96-well plate and incubated at 37°C for 24 h. Afterward, the medium was aspired and a new RPMI-1640 medium with or without EP (0.5, 1, 2, 4 and 8xMIC) was added and incubated at 37°C for further 24 h. Finally, the effect of EP on matured biofilms was examined under an inverted microscope.

### Biofilms formation in silicone tubes and analysis by scanning electron microscope

Biofilms were allowed to generate in a silicone tube system under a dynamic condition. Tap water from a public water distributor was used as the source of biofilm-forming microorganisms. Briefly, at a flow rate of 200 mL/h tape water was pumped through a silicone tube with an inner diameter of 2 mm of (Carl Roth, Karlsruhe, Germany) at 30°C for 14 days. Afterward, 3 cm-pieces of the silicone tube were cut before treatment (control) and the remaining tube was immersed in 50 mM phosphate-buffered EP, EL and Pen/Strep (100 units/μg/mL corresponding to 0.28 and 0.17 mM for Pen and Strep, respectively) solutions for further 24 h. Next, the cut piece were emptied and sealed at both ends. Subsequently, the outer surface was treated with 70% ethanol and rinsed with sterile distilled water. Afterward, the tubes were cut longitudinally over the entire surface into two pieces and then dipped into LB broth followed by detachment of the biofilm through a vigorous vortex for 2–3 min. Subsequently, 0.1 mL of a 1:10 diluted suspension was seeded onto LB agar plates and incubated at 37°C for 5 days. To determine the time-dependency the silicone tubes were analysed at different time points e.g. at 0, 4, 8 and 24 h.

### Scanning electron microscopy

To examine the ultrastructural morphology of the biofilm, scanning electron microscopy was performed. First, 10 mm long pieces were cut from silicone tubes containing biofilms treated with 50 mM EP. Second, to prevent drying each piece was instantly submerged in 0.1 M PBS and cut longitudinally, resulting in two halfpipe-shaped specimens. Specimens were washed in 0.1 M PBS, pH 7.2, fixed with 2.5% glutaraldehyde in 0.1 M cacodylate buffer, at25°C for 1 h. Then, the specimens were washed three times in the same buffer and post-fixed with 1% osmium tetroxide in 0.1 M cacodylate buffer for 2 h and dehydrated in an increasing series of ethanol (30%, 50%, 70%, 90% and absolute ethanol) for 30 min each. After critical-point-drying (CPD 030, Bal-Tec, Liechtenstein) specimens were mounted on SEM sample carrier and coated with gold-palladium (90/10) using a sputter coater (MED 020, Bal-Tec, Liechtenstein). The biofilm was inspected at different magnifications with a scanning electron microscope (LEO 1430, Zeiss, Oberkochen, Germany).

### Data analysis

At first MIC data was entered to excel spreadsheet version 2013 and descriptive statistic was employed to demonstrate the distribution of isolates. Student’s *t*-test and one-way ANOVA were used to analyze the tested microbes using GraphPad Prism version 5 (San Diego, USA). The data were expressed as mean ± SEM. *P*-value <0.05 was considered as statistically significant.

## Results

**Fig 2:**
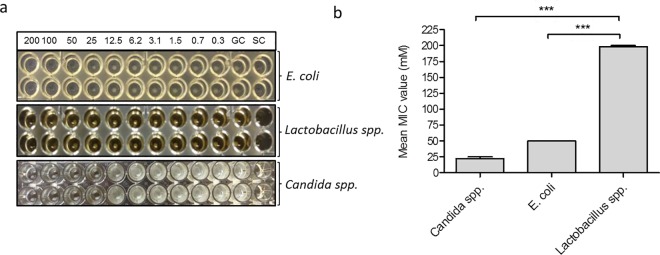
Effect of ethyl pyruvate on growth of planktonic pathobionts. (a) Representative diagram of MIC determination for *E*. *coli*, *Lactobacillus spp*. and *Candida spp*. in 96-well plate. The final EP concentration ranging from 0.39 to 200 mM; growth control (GC), and sterility control (SC). White spots in the wells depict the growth of tested isolates. (b) Selective anti-microbial activity of EP against pathobionts, e.g. *Candida spp*. (n = 108), *Escherichia coli* (n = 8), and *Lactobacillus spp*. (n = 31) (***P< 0.0001).

**Pathobiont-selective activity of ethyl pyruvate against microbes of vaginal specimens** *Candida spp*. are key pathobionts in vulvovaginal candidiasis that results mainly from the loss of normal Lactobacillus-predominant vaginal microbiota that is mostly due to uncontrolled and frequent antibiotic intake [19]. We have chosen representative microorganisms of the vaginal microbiota for studying the effect of EP on individual species. The number and type of microbial isolates identified from vaginal specimen were subsequently used to determine the growth inhibitory activity of EP. Altogether, 147 isolates were obtained from vaginal specimens. Among these, Candida isolates were the predominant, which accounted for 73.5% of the total isolated microbes followed by *Lactobacillus spp*. (21%) and *E*. *coli* (5.4%) (Table 1). The minimum inhibitory concentration of EP (MIC_EP_) against the depicted isolates of *Candida albicans* (n = 57), *Candida tropicalis* (n = 10) and *Candida parapsilosis* (n = 15) were similar (25±0 mM), irrespective of the genetic make-up of the strains of each species. A slightly higher concentration of EP was required to inhibit the growth of *Candida glabrata* (n = 11) (28.41±4.87mM). Among the tested yeasts isolates, *Candida krusei* (n = 5) was the most susceptible one with a MIC_EP_ value of 12.5±0 mM. Furthermore, the MIC_EP_ values of all yeast isolates except *Candida glabrata* were similar to the minimum fungicidal concentration (MFC_EP_) value. Higher MIC_EP_ values were ascertained for *Escherichia coli* isolates (50±0 mM). In contrast, the MIC_EP_ values for *Lactobacillus spp*., particular *Lactobacillus jensenii*, *Lactobacillus acidophilus/gasserii*, *Lactobacillus crispatus*, *Lactobacillus rhamnosus*, *Lactobacillus casei/paracasei*, and *Lactobacillus delbrueckii* were very high, ranging from 192.86±26.73 mM to ≥ 200 mM, when compared with the ones for pathogenic microbes. Fascinatingly, greater minimum bactericidal concentration (MBC_EP_) values (>200 mM) of *Lactobacillus spp*. were observed, signifying the harmlessness of EP for the beneficial microbiota of the host. Generally, potentially pathogenic microbes were more susceptible to EP, whilst the beneficial isolates resisted (p<0.0001) (Fig 2A and 2B).

**Table 1:** Distribution of identified vaginal isolates and their MIC, MBC and MFC values.

Microorganisms	Species	Number of isolates (%)	MIC (Mean±SD)	MFC/MBC (Mean±SD)
*Candida* species	*Candida albicans*	57 (38.8)	25±0	25±0
	*Candida glabrata*	11 (7.5)	28.41±4.87	35.83±9.91
	*Candida tropicalis*	10 (6.8)	25±0	25±0
	*Candida parapsilosis*	15 (10.2)	25±0	25±0
	*Candida krusei*	5 (3.4)	12.5±0	12.5±0
	Others	10 (6.8)	18.75±6.59	25±14.43
*Lactobacillus* species	*Lactobacilus jensenii*	14 (9.5)	192.86±26.73	>200
	*Lactobacillus acidophilus/gasserii*	7 (4.76)	200±0	>200
	*Lactobacillus rhamnosus*	2 (1.36)	>200	>200
	*Lactobacillus casei/paracasei*	2 (1.36)	>200	>200
	*Lactobacillus crispatus*	5 (3.4)	200±0	>200
	*Lactobacillus delbrueckii*	1 (0.7)	200	200
Gram negative bacteria	*Escherichia coli*	8 (5.44)	50±0	100±0
Total		147		

Clinical isolates were cultured onto appropriate culture media and identified by Matrix-assisted laser desorption ionization time-of-flight (MALDI-TOF). Minimum inhibitory and minimum bactericidal or fungicidal concentrations of ethyl pyruvate (EP) were determined by broth microdilution techniques. The results are expressed in mean ± SD (n = 3).

### Ethyl pyruvate inhibits the growth of a wide panel of fungal and bacterial pathobionts

**Fig 3:**
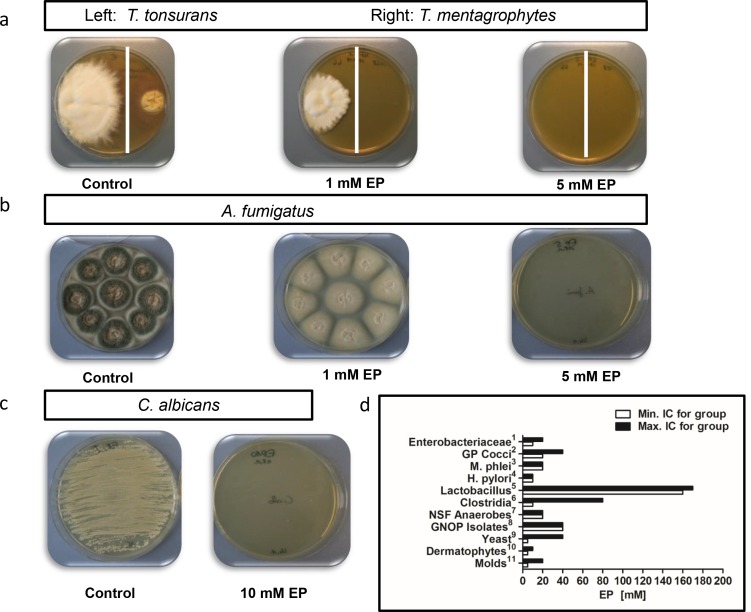
Inhibition by ethyl pyruvate of the growth of a wide panel of fungal and bacterial pathobionts. (a) Cultures of *Trichophyton mentagrophytes* (left, white) and *Trichophyton tonsurans* I (right, yellow) were treated with different concentration of EP (0, 1 and 5 mM) for 24 hours. Cultures of *Aspergillus fumigatus* (b) and *Candida albicans* (c) were treated by EP at 0, 1, 5, and 10 concentrations (mM). (d) Minimum (Min) and maximum (Max) inhibitory concentration of EP for different bacteria, fungi and moulds (S1 Fig).

Fungal infection caused by moulds, dermatophytes and yeasts have been health problem of both healthy and immunocompromised individuals [20]. Notably, Dermatophytes and *Malassezia spp*., have been the leading etiologic agents for skin diseases, resulting in a significant morbidity, discomfort, and disfigurement [21]. Furthermore, *Aspergillus fumigatus*, is the most frequent cause of invasive fungal infection primarily in the lung, causing a high mortality and morbidity in immunocompromised individuals [22]. In this line, we have extended our survey and analyzed the growth inhibitory potential of EP against a number of fungal pathobionts including Dermatophytes and moulds. Most Dermatophytes could be growth-inhibited by least 5mM EP with the exception of *Microsporum gypseum*, which was dumped at 10 mM EP (Table 2). A representative example is shown for *Trichophyton mentagrophytes*, *Trichophyton tonsurans*, *Aspergillus fumigatus* and *Candida albicans* (Fig 3A–3C). Surprisingly, the growth of *Aspergillus fumigatus* was completely inhibited at 5mM EP (Fig 3B). We further evaluated the growth inhibitory potential of EP against clinically relevant pathobionts such as non-spore forming anaerobes, Gram negative oxidase positive bacteria, *Clostridium spp*., *Helicobacter pylori*, Gram positive cocci, and *Salmonella spp*. Accordingly, the minimum inhibitory concentration of EP against these pathobionts was ranged from 10 to 40 mM (Fig 3D).

**Table 2:** Effect of ethyl pyruvate on dermatophytes.

Species		Concentration (mM)	Days				
			2	4	6	14	21
*Trichophyton mentagrophytes*	Control	0	+	+	+	+	+
	EP	1	-	-	-	+	+
		5	-	-	-	-	-
		10	-	-	-	-	-
*Trichophyton interdigitale*	Control	0	+	+	+	+	+
	EP	1	-	-	-	(+)	+
		5	-	-	-	-	-
		10	-	-	-	-	-
*Microsporum gypseum*	Control	0	+	+	+	+	+
	EP	1	+	+	+	+	+
		5	-	-	-	-	+
		10	-	-	-	-	-
*Microsporum canis*	Control	0	+	+	+	+	+
	EP	1	-	-	(+)	+	+
		5	-	-	-	-	-
		10	-	-	-	-	-
*Trichophyton rubrum* I	Control	0	+	+	+	+	+
	EP	1	-	-	-	(+)	+
		5	-	-	-	-	-
		10	-	-	-	-	-
*Trichophyton rubrum* II	Control	0	+	+	+	+	+
	EP	1	-	-	(+)	+	+
		5	-	-	-	-	-
		10	-	-	-	-	-
*Trichophyton tonsurans* I	Control	0	-	+	+	+	+
	EP	1	-	-	-	(+)	+
		5	-	-	-	-	-
		10	-	-	-	-	-
*Trichophyton tonsurans* II	Control	0	-	-	-	+	+
	EP	1	-	-	-	-	-
		5	-	-	-	-	-
** **		10	-	-	-	-	-

Culture media containing ethyl pyruvate (EP) at different concentrations and control media without EP were prepared. Standardized microbial suspension was poured onto plates and incubated for certain time at appropriate temperature and followed by microbial growth investigation and recording. Accordingly, + = growth, (+) = growth doubtful,– = no growth.

### Ethyl pyruvate inhibits formation of *Candida albicans* biofilms and promotes resolution of mature biofilms

**Fig 4:**
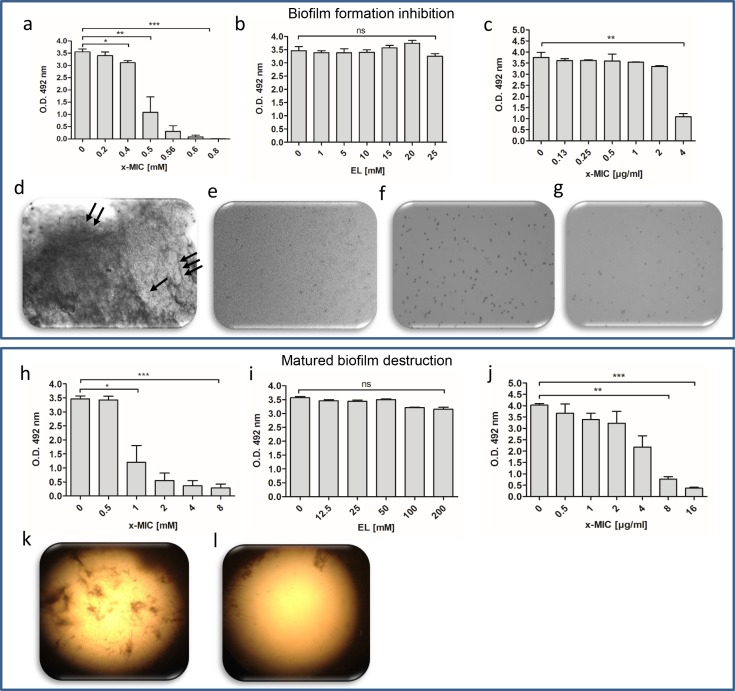
Ethyl pyruvate inhibits formation and dispersion of *Candida albicans* biofilms. Biofilms were grown in a 96-well tissue culture plates in the presence of ethyl pyruvate (EP) (a), ethyl lactate (EL) (b) and Amphotericin B (AmpB) (c). The corresponding MIC-values were obtained from planktonic cell studies. Fluorescence microscopic analysis revealed the effect of EP on *Candida albicans* biofilms formation (d-g). Non-treated biofilms showing dense yeast cells (single arrow) and hyphae (triple arrow) and the extracellular matrix (double arrow) casing the yeast cells (d). Treatment of cells by 10, 25, and 50 mM EP is shown in e, f and g, respectively. Calcofluor staining was used to generate the images at x10 magnification using fluorescence microscopy. Dispersion of matured *Candida albicans* biofilm by EP (h), EL (i) and AmpB (j). An inverted light microscopic analysis of non-treated pre-formed *Candida albicans* biofilms shows filamentous biofilms containing both hyphae and yeast cells (k). Destructed biofilm by EP (4xMIC) displays no cells and hyphae (l). Results are mean ± standard error of the mean of three independent experiments. *p<0.05; **p<0.01, ***p<0.001.

Beside vaginal, oral candidiasis and additional superficial infections, systemic infection by *Candida spp*. is linked with serious life-threatening conditions [23]. The crucial virulent factors of *Candida albicans* is its ability to grow in a variety of morphological forms, ranging from unicellular budding yeast to filamentous form, that are able to invade tissues and overcome immune response [24]. Moreover, particularly *Candida albicans* are known to form biofilms on implants, mucosal surfaces and vaginal epithelial cells [6]. The inhibitory activity of EP against *Candida albicans* planktonic cells as shown in Fig 2B prompted us to study the effect of EP on *Candida albicans* biofilm formation and resolution *in vitro* (Fig 4). EP inhibited formation of *Candida albicans* biofilm in a dose-dependent manner already at sub-inhibitory concentrations indicated by low MIC_EP_ values (P = 0.02 for 0.4 and 0.5xMIC_EP_) (Fig 4A). At concentrations of 0.8xMIC_EP_ biofilm formation was almost completely blocked (P <0.0001). In contrast to EP, EL had no effect on *Candida albicans* biofilm formation indicating the requirement of certain structural elements in the molecule to be effective (Fig 4B). For comparison, biofilm formation could not be inhibited by the standard antifungal drug Amphotericin B (AmpB) at least at concentrations equivalent to 2xMIC_AmpB_ (P = 0.01) (MIC _AmpB_ = 0.38 μg/mL) (Fig 4C). Fluorescence microscope analysis revealed complex and dense yeast cells as well as hyphae encased in a matrix in non-treated samples (Fig 4D). Clearly, EP-treated samples were unable to develop biofilms (Fig 4E–4G) and yeast cells number vanished in a concentration dependent manner.

EP exerted inhibitory activity also against pre-formed *Candida albicans* biofilm. After incubation of matured biofilms with EP for 24 h, a decided destruction of pre-formed biofilms was recorded. Interestingly, EP significantly decreased metabolic activity of pre-formed biofilms already at 1xMIC_EP_ concentration (Fig 4H), which is not seen with the conventional antifungal drug AmpB (Fig 4J). This is further witnessed by the finding that high concentrations of AmpB (8x and 16xMIC_AmpB_) had to be applied to obtain a significant reduction of pre-formed biofilms. Again, EL displayed no effect on matured *Candida albicans* biofilms (Fig 4I). These results were confirmed by inverted microscopy analysis clearly showing a dense multilayered network of yeast cells and hyphae in non-treated matured biofilms (Fig 4K). The complete destruction of pre-formed biofilms including dissolution of the matrix upon EP treatment is presented in Fig 4L.

### Ethyl pyruvate disperses the matrix of biofilms prepared in silicone tubes under dynamic fluid condition

**Fig 5:**
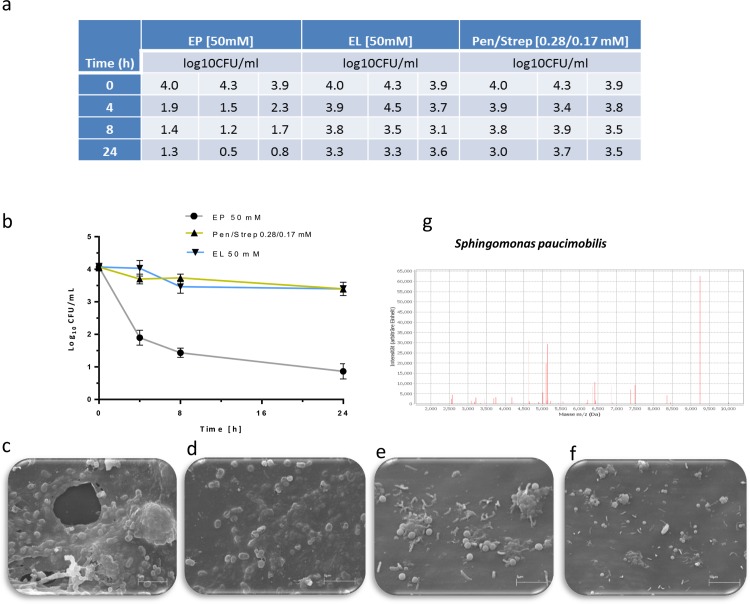
Destruction by ethyl pyruvate of biofilms prepared under dynamic fluid condition. (a) Colony forming units derivable from biofilms before and after treatment with ethyl pyruvate (EP) in silicone tubes. (b) Treatment of matured biofilms in silicone tubes by 50 mM EP and 50 mM ethyl lactate (EL) as well as Penicillin/Streptomycin (0.28/0.17 mM) as a control. Destruction of biofilm in the presence of antibacterial compounds was evaluated at different time intervals. Scanning electron microscopy clearly shows well-structured matured biofilm, containing microorganisms encased in extracellular matrix (c, d). EP treatment (50mM) displays few cells, cell debris and dissolution of extracellular matrix (e, f). Representative profile of MALDI-TOF mass spectrum of the identified *Sphingomonas paucimobilis* from the silicone tube biofilm (g).

Bacterial pathobionts are capable of forming complex biofilms that potentially results in several infections. Explicitly, cystic fibrosis, native valve endocarditis, otitis media, periodontitis chronic prostatitis are appeared to be due to bacterial biofilms [5]. Remarkably, bacterial biofilm infections are from both Gram-positive and Gram-negative bacteria that might be originated from the skin, from contact with other individuals or from tap water [25]. We have developed bacterial biofilms under dynamic fluid condition using tap water as a source and treated them with EP, EL, and Penicillin/Streptomycin (Pen/Strep). The experimental results demonstrate a 3xlog_10_ reduction of the number of colony-forming units of the biofilms upon exposure to 50 mM of EP (Fig 5A). In contrast, EL and Pen/Strep only marginally reduced the colony-forming units from 4xlog_10_ to 3.3 and 3xlog_10_ CFU/mL, respectively (Fig 5B). Moreover, biofilm destruction by EP became apparent already after 4 h of treatment. Inspection of biofilm by scanning electron microscopy revealed a very dense and complex structure of matured biofilms showing poly-microbial communities encased within the matrix of extracellular polymeric substances (Fig 5C and 5D). After treatment of the biofilm with EP the number of encased microbes tremendously decreased and only few vital cells were left surrounded by cell debris. Surprisingly, EP distorted the biofilm morphology not only by cell growth inhibition but also by dissolution of the biofilm matrix (Fig 5E and 5F). Notably, no matrix destruction was observed in case of EL. This enables EP to become a potent agent to inhibit microbes’ adhesion, biofilm colonization, cell proliferation and to promote matrix destruction.

### Ethyl pyruvate ignores resistance of microbes against a common drug

**Fig 6:**
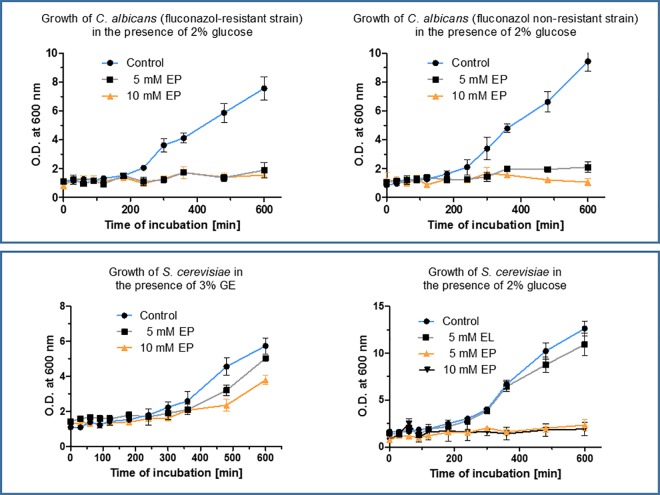
Growth inhibition of fluconazole-resistant and susceptible *Candida albicans* by ethyl pyruvate. The growth of both resistant and non-resistant *Candida* isolates in the presence of glucose was inhibited by EP (a, b), whereas when gluconeogenesis was initiated with 3% glycerol/ethanol (GE) medium (c, d).

In invasive fungal infections, *Candida spp*., mould and *Aspergillus spp*. are absolutely the dominating pathogens. The fluconazole-susceptible clinical isolate, *Candida albicans* (CA 8668), showed a MIC of 16 μg/mL fluconazole and hence is a susceptible dose-dependent (SDD) strain according to the CLSI guidelines [26]. The fluconazole-resistant strain, *Candida albicans* (CA II64), displayed a MIC of 64 μg/mL. Both strains were tested for their capability to grow in response to EP treatment (Fig 6A and 6B). In the presence of 2% glucose in the medium EP effectively inhibited the growth of the fungi irrespective of their degree of sensitivity to fluconazole. Recovery tests that were performed by long-term incubation (two weeks) of the strain treated before with 20 mM EP for two 24 hours showed no cell growth at all, indicating complete cell killing. When yeast cells were grown in medium favouring gluconeogenesis (3% glycerol/ethanol (GE) medium) instead of glycolysis (2% glucose medium) the efficacy of EP was slashed (Fig 6C and 6D). This indicates that EP primarily inhibits growth of those cells that exhibit a high glycolytic throughput. On the other hand, it offers the possibility to boost the inhibitory action of EP by co-stimulation of the glycolysis.

## Discussion

In humans bacteria, fungi, virus and archaea live together in communities (microbiota), and colonize different parts of the body, such as the gut, oral cavity, skin, eyes and vagina [27]. Growing evidence supports the notion that alteration of the microbiota equilibrium has a strong influence on health and disease in humans [28]. Factors contributing to this alteration are diet, genetics, environment, antibiotic intake and age [29]. This may consequently result in conditions of dysbiosis that will end up in either a reduction in the number of symbionts, an imbalance between the distinct symbionts and/or an increase in the number of pathobionts [30]. That exigently calls for the development of pathobiont-targeted anti-microbial agents.

Moreover, many microbial pathogens are capable of forming biofilms resulting in persistent infections that are difficult to treat and capable of evading the host immune system [25]. Biofilms are formed when bacteria, fungi and/or algae adhere to biotic or abiotic surfaces encase themselves in an extracellular matrix and grow as sessile communities [5]. Biofilm developmental stages comprise at least three major events: (i) initial attachment, (ii) biofilm maturation, and (iii) dispersal [31]. After surface attachment biofilm maturate through cell division and the production of the extracellular polymeric matrix. Biofilm matrix varies between strains, but in general can contain host factors, polysaccharide, proteins, and extracellular DNA. Mostly, negatively charged polymeric sugars and DNA are bridged by Ca^++^ ions which stabilize the matrix architecture. In order to coordinate the formation of biofilms, microorganisms use a cell-to-cell communication system called quorum sensing (QS). It involves signal molecules called auto-inducers that are released by the microbes themselves such as peptides (Gram-positive bacteria) or lipid-based molecules (Gram-negative bacteria) or farnesol (fungi) [32]. Once the population reached a specific density or threshold, expression of certain genes such as virulence factors and adhesion proteins can occur. Biofilm-related infections may account for more than 65% of all microbial infections in the human body [33].

Methods employed for preventing and eliminating biofilms are limited in their efficacy on mature biofilms. Despite this a number of anti-biofilm formulations and technologies that include ethylenediaminetetraacetic acid (EDTA) [34], surface-coated nanoparticles [35], small molecular organic compounds [36], weak acids [37], silver [38] and monoclonal antibodies [39] have demonstrated efficacy on *in vitro* biofilms. Still, there is a need for agents that act against both planktonic cells and biofilm and exert minimal tissue-irritating effects.

As learned from cancer research, EP, when used in comparable concentrations inhibited tumor cells growth effectively, most probably by targeting enzymes of the glycolytic pathway [11, 13, 14]. Contact with EP induced necroptosis of cancer cells primarily by induced depletion of ATP and the formation of the toxic methylglyoxal. Since the glycolytic chain is highly conserved in almost all microbes investigated it is likely to assume similar mechanism of action of EP as in mammalian cells. This is corroborated by the findings that EP is less toxic to cells when they gain their energy from gluconeogenesis instead of glycolysis (Fig 6). This, on the other hand, means that the toxicity of EP can be enhanced by metabolic activation of microbes or when microbes growth under nutrient-enriched condition. From the current point of knowledge, EP has the edge over other anti-biofilm agents for several reasons: (i) no side effects could be seen in clinical studies so far [40], (ii) it is tissue protective [10], (iii) it is inhibitory to a broad spectrum of pathobionts (bacteria, fungi, moulds, parasites) and (iv) harmless to symbionts (*Lactobacillus spp*.), (v) there is a low chance of resistance development as EP is aimed at multiple targets (vi) it inhibits biofilm adhesion and maturation, (vii) it dissolutes the biofilm matrix, (viii) it is environmentally safe [41].

The surprising different anti-microbial activity of EP and EL awaits explanation. Chemically, they differ only from two protons, which consequently allow EP to configure a dicarbonyl structure [10]. This structural element is a prerequisite for inhibiting the enzymes GLO1 and PK as EL revealed no effect at all. It may also disclose why many polyphenols such as curcumin and resveratrol were reported to have anti-microbial activities [42, 43].

The potency of EP to dissolute pre-formed biofilm has to be emphasized. It most probably relates to its property to chelate divalent ions such as Ca^++^ ions due to its dicarbonyl structure. Targeting Ca^++^ ions in biofilms by EDTA has been proposed as treatment of infected wounds [34].

Still a matter of discussion that remains is the millimolar concentration of EP needed to assure inhibitory effects. Therefore, it is currently questionable for using it as a systemic drug medication. However, an improvement of its activity could be anticipated by chemically manipulating the core structure. Nevertheless, infusion of EP has already been applied in clinical studies and no side effects of EP in humans were reported [40]. In fact most microbial infections are located on the skin and mucosa such as in the vagina and gut. In this line, EP-grafted dressings or polymers with a sustained release of EP into the wound bed or inflamed gut could be a new option for wound care especially in surgery, diabetes and bowel diseases [44, 45].

It is suggested that chronic inflammation as typically observed in intestinal bowel disease involves a combination of *microbial* inflammation as well as *sterile* inflammation, the latter induced by components of fragmented cells such as DNA or nuclear proteins like HMGB1. Notably, EP is capable to act against both causes [46].

As far as bacterial vaginosis biofilms are concerned vaginal tampons containing EP could be used to reconstitute the vaginal microbiota. This is all the important as preservation of *Lactobacillus spp*. in the mucosal fluids is a central issue in case of treatment with antibiotics.

The devastating effects of antibiotic resistance may look us into a disquieting prospect, which threatens our dwindling antibiotic arsenal. Antibiotic resistance is a global problem leading to an increased economic and human cost in lives [47]. Notably, these changes in resistance rates include increasing detection of extended spectrum beta-lactamase (ESBL)-production in *Enterobacteriaceae*, increasing multidrug-resistance in *P*. *aeruginosa* and *Acinetobacter spp*., Methillicin-resistant *Staphylococcus aureus* (MRSA), as well as in *Candida* species. In addition, Enterohemorrhagic *Escherichia coli* (EHEC), a food born human pathogen, has been accounting for outbreaks of bloody diarrhea and haemolytic uraemic syndrome especially in young children and elderly worldwide [48]. In the present study we have shown for the first time that EP inhibited microbes known to be resistant to most available drugs in the clinics.

Apart from clinical aspect, the applicability of EP could be extended to industrial level. As biofilm forming microorganisms are highly prevalent in dead ends, corners, cracks, crevices, gaskets, valves, medical devices and the joints of stainless steel equipment used in the dairy manufacturing plants [49]. The removal of biofilms and planktonic cells within production machinery in the paper, safety and hygienic food packaging industry [50], cooling water circuits e.g. in nuclear power stations, and drinking water manufacturing systems can be critical for the safety and efficacy of those processes [51].

In summary, EP revealed a selective anti-microbial action against both potentially pathogenic fungal and bacterial planktonic cells and complex biofilms. This intensifies the potential of the compound specifically to be used as an anti-microbial agent against disease causing pathogens without affecting beneficial bacteria.

## Supporting Information

S1 Fig Description of microbes tested in the gowth inhibitory assay. 1: Enterobacteriaceae: *Salmonella agona*, *Salmonella* B(O:4), *Salmonella derby*, *Salmonella brandenburg*, *Salmonella corvallis*, *Salmonella london*, *Salmonella ohio*, *Salmonella goldcoast*, *E*. *coli Nissle*, Enterohaemorrhagic *E*. *coli* (EHEC); 2: Gram positive (GP) cocci: *Staphylococcus aureus*, *Streprococcus dysgalactiae ssp*. *dysgalactiae*, Methicillin-resistant *Staphylococcus aureus* (MRSA); 3: *Mycobacterium* (*M*) *phlei*. 4: *Helicobacter (H) pylori* 5: *Lactobacillus*: *Lactobacillus paracasei ssp*. *paracasei*, *Lactobacillus brevis*, *Lactobacillus buchneri*, *Lactobacillus harbinensis*, *Lactobacillus plantarum*; 6: *Clostridia*: *Clostridium cutyricum*, *Clostridium*. *baratii*, *Clostridium difficile*, *Clostridium chauvoei*, *Clostridium sordellii*, *Clostridium septicum*, *Clostridium bifermentans*, *Clostridium sporogenes*, *Clostridium botulinum* type A-E, *Clostridium novyi*, *Clostridium perfringens* type B-D, *Clotridium tetanomorphum*, *Clostridium tetani*; 7: Non-spore forming (NSF) anaerobes: *Porphyromonas gingivalis*, *Prevotella intermedia*; 8: Gram- negative oxidative positive (GNOP) isolates: *Pseudomonas aeruginosa*; *Campylobacter jejuni*, *Campylobacter coli*. 9: Yeast: *Candida albicans*, *Candida parapsilosis*, *Candida kruzei*, *Candida tropicalis*, *Candida valida*, *Candida lusitaniae*, *Candida pulcherrima*, *Candida glabrata*, *Candida zeylanoides*, *Candida boidinii*, *Candida kefyr*, *Candida allociferii*, *Candida cacaoi/Pichia farinosa*, *Candida africana*, *Candida pararugosa*, *Candida colliculosa*, *Candida guilliermondii*, *Candida lipolytica*, *Candida haemulonii*, *Candida dubliniensis*, *Candida norvegensis*, *Candida thermophilia*, *Candida slooffiae*, *Sporomolomyces salmonicolor*, *Magnusiomyces capitatus*, *Rhodotorula glutinis*, *Rhodotorula mucilaginosa*, *Trichosporon ovoides*, *Metschnikowia reukaufii*, *Malassezia pachydermatis*, *Cryptococcus laurentii*; *Trichosporon debeurmanniam*, 10: Dermatophytes: *Trichophyton mentagrophytes*, *Microsporum gypseum*, *Trichophyton rubrum*, *Microsporum canis*, *Trichophyton interdigitale*, *Trichophyton tonsurans*, *Trichophyton mentagrophtes*, *Microsporom audouinii*; 11: Molds: *Aspergillus fumigatus*, *Aspergillus flavus*, *Mucor spp*., *pseudallescheria*, *Trichoderma spp*., *Aspergillus niger*, *Altenaria spp*.; Min. IC = Minimum Inhibitory Concentration for the group; Max. IC = Maximum Inhibitory Concentration for the group.
(PDF)
