# Supplementary material for: Ethyl Pyruvate: An Anti-Microbial Agent that Selectively Targets Pathobionts and Biofilms
Source: PLoS One. 2016 Sep 22;11(9):e0162919. doi: 10.1371/journal.pone.0162919 (PMC5033407; doi:10.1371/journal.pone.0162919)
Supplement: S1 Fig — 1: Enterobacteriaceae: Salmonella agona, Salmonella B(O:4), Salmonella derby, Salmonella brandenburg, Salmonella corvallis, Salmonella london, Salmonella ohio, Salmonella goldcoast, E. coli Nissle, Enterohaemorrhagic E. coli (EHEC); 2: Gram positive (GP) cocci: Staphylococcus aureus, Streprococcus dysgalactiae ssp. dysgalactiae, Methicillin-resistant Staphylococcus aureus (MRSA); 3: Mycobacterium (M) phlei. 4: Helicobacter (H) pylori 5: Lactobacillus: Lactobacillus paracasei ssp. paracasei, Lactobacillus brevis, Lactobacillus buchneri, Lactobacillus harbinensis, Lactobacillus plantarum; 6: Clostridia: Clostridium cutyricum, Clostridium. baratii, Clostridium difficile, Clostridium chauvoei, Clostridium sordellii, Clostridium septicum, Clostridium bifermentans, Clostridium sporogenes, Clostridium botulinum type A-E, Clostridium novyi, Clostridium perfringens type B-D, Clotridium tetanomorphum, Clostridium tetani; 7: Non-spore forming (NSF) anaerobes: Porphyromonas gingivalis, Prevotella intermedia; 8: Gram- negative oxidative positive (GNOP) isolates: Pseudomonas aeruginosa; Campylobacter jejuni, Campylobacter coli. 9: Yeast: Candida albicans, Candida parapsilosis, Candida kruzei, Candida tropicalis, Candida valida, Candida lusitaniae, Candida pulcherrima, Candida glabrata, Candida zeylanoides, Candida boidinii, Candida kefyr, Candida allociferii, Candida cacaoi/Pichia farinosa, Candida africana, Candida pararugosa, Candida colliculosa, Candida guilliermondii, Candida lipolytica, Candida haemulonii, Candida dubliniensis, Candida norvegensis, Candida thermophilia, Candida slooffiae, Sporomolomyces salmonicolor, Magnusiomyces capitatus, Rhodotorula glutinis, Rhodotorula mucilaginosa, Trichosporon ovoides, Metschnikowia reukaufii, Malassezia pachydermatis, Cryptococcus laurentii; Trichosporon debeurmanniam, 10: Dermatophytes: Trichophyton mentagrophytes, Microsporum gypseum, Trichophyton rubrum, Microsporum canis, Trichophyton interdigitale, Trichophyton tonsurans, Trich [file pone.0162919.s001.pdf]

Supporting Information

S1 Fig 3d. Description of microbes tested in the gowth inhibitory assay

1: Enterobacteriaceae: *Salmonella agona*, *Salmonella* B(O:4), *Salmonella derby*, *Salmonella brandenburg*, *Salmonella corvallis*, *Salmonella london*, *Salmonella ohio*, *Salmonella goldcoast*, *E. coli Nissle*, Enterohaemorrhagic *E. coli* (EHEC); 2: Gram positive (GP) cocci: *Staphylococcus aureus*, *Streprococcus dysgalactiae ssp. dysgalactiae*, Methicillin-resistant *Staphylococcus aureus* (MRSA); 3: *Mycobacterium* (*M*) *phlei*. 4: *Helicobacter (H) pylori* 5: *Lactobacillus: Lactobacillus paracasei ssp. paracasei*, *Lactobacillus brevis*, *Lactobacillus buchneri*, *Lactobacillus harbinensis*, *Lactobacillus plantarum*; 6: *Clostridia: Clostridium cutyricum*, *Clostridium. baratii*, *Clostridium difficile*, *Clostridium chauvoei*, *Clostridium sordellii*, *Clostridium septicum*, *Clostridium bifermentans*, *Clostridium sporogenes*, *Clostridium botulinum* type A-E, *Clostridium novyi*, *Clostridium perfringens* type B-D, *Clotridium tetanomorphum*, *Clostridium tetani*; 7: Non-spore forming (NSF) anaerobes: *Porphyromonas gingivalis*, *Prevotella intermedia*; 8: Gram- negative oxidative positive (GNOP) isolates: *Pseudomonas aeruginosa*; *Campylobacter jejuni*, *Campylobacter coli*. 9: Yeast: *Candida albicans*, *Candida parapsilosis*, *Candida kruzei*, *Candida tropicalis*, *Candida valida*, *Candida lusitaniae*, *Candida pulcherrima*, *Candida glabrata*, *Candida zeylanoides*, *Candida boidinii*, *Candida kefyr*, *Candida allociferii*, *Candida cacaoi/Pichia farinosa*, *Candida africana*, *Candida pararugosa*, *Candida colliculosa*, *Candida guilliermondii*, *Candida lipolytica*, *Candida haemulonii*, *Candida dubliniensis*, *Candida norvegensis*, *Candida thermophilia*, *Candida slooffiae*, *Sporomolomyces salmonicolor*, *Magnusiomyces capitatus*, *Rhodotorula glutinis*, *Rhodotorula mucilaginosa*, *Trichosporon ovoides*, *Metschnikowia reukaufii*, *Malassezia pachydermatis*, *Cryptococcus laurentii*; *Trichosporon debeurmanniam*, 10: Dermatophytes: *Trichophyton mentagrophytes*, *Microsporum gypseum*, *Trichophyton rubrum*, *Microsporum canis*, *Trichophyton interdigitale*, *Trichophyton*

tonsurans, *Trichophyton mentagrophtes*, *Microsporom audouinii*; 11: Molds: *Aspergillus fumigatus*, *Aspergillus flavus*, *Mucor spp*., *pseudallescheria*, *Trichoderma spp*., *Aspergillus niger, Altenaria spp*.; Min. IC = Minimum Inhibitory Concentration for the group; Max. IC = Maximum Inhibitory Concentration for the group.
